# The relationship between smartphone addiction and shyness among medical college students: The mediating effect of self-acceptance

**DOI:** 10.1097/MD.0000000000050906

**Published:** 2026-09-25

**Authors:** Zhimin Yin, Rong He, Renjie Chang, Qin Liu

**Affiliations:** a School of Nursing, Yunnan University of Traditional Chinese Medicine, Kunming, Yunnan, China; b The First Affiliated Hospital of Yunnan University of Traditional Chinese Medicine (Yunnan Provincial Hospital of Traditional Chinese Medicine), Kunming, Yunnan, China.

**Keywords:** mediating effect, self-acceptance, shyness, smartphone addiction

## Abstract

Smartphone addiction has become a widespread issue affecting the mental health of medical students. Shyness, a common personality trait, and self-acceptance have not been fully explored for their associative relationships with smartphone addiction among this population. This study aimed to explore the relationships among shyness, self-acceptance, and smartphone addiction, and to examine the mediating role of self-acceptance in these relationships. We adopted a cross-sectional survey, using the Smartphone Addiction Scale-Short Version, Shyness Scale, and Self-Acceptance Questionnaire, to conduct a questionnaire survey among 756 undergraduate students at a medical school in southwestern China. Results showed that shyness was significantly positively correlated with smartphone addiction (*R *= 0.312, *P *< .001) and significantly negatively correlated with self-acceptance (*r *= −0.621, *P *< .001). Self-acceptance was also significantly negatively correlated with smartphone addiction (*r *= −0.301, *P < *.001). Mediation analysis indicated that self-acceptance partially mediated the relationship between shyness and smartphone addiction, accounting for 34.1% of the total effect. Shyness was associated with lower self-acceptance, which in turn was associated with a higher level of smartphone addiction.

## 1. Introduction

In the digital age, smartphones have become indispensable tools in the daily learning and lives of college students. However, overuse of smartphones is closely associated with smartphone addiction.^[1]^ As a form of behavioral addiction, smartphone addiction is associated with adverse psychosocial outcomes, including anxiety, depression, sleep disorders, and declining academic performance.^[2–4]^ Medical students, in particular, face unique challenges of high academic pressure and the need for effective interpersonal communication in their future careers.^[5,6]^ They have a smartphone addiction rate of 41.93%, significantly higher than the 36.6% rate among general college students.^[7,8]^ Smartphone addiction may exacerbate their academic stress and social difficulties,^[9,10]^ making it crucial to investigate the influencing factors and mechanisms of smartphone addiction among medical students.

Shyness is a personality trait characterized by social discomfort, anxiety about evaluation, and avoidance of social interactions.^[11]^ For medical students whose future careers require frequent, high-quality doctor–patient communication, this trait poses a dual challenge. On 1 hand, to avoid the uncertainties of real-world social interactions, shy individuals tend to use smartphones to create secure virtual social environments, thereby replacing and limiting real-life social engagement.^[12]^ Long-term reliance on virtual socialization may deteriorate real-world social skills, forming a vicious cycle of “shyness to virtual social dependence to real-world social avoidance,” ultimately increasing the risk of smartphone addiction.^[13]^ Additionally, research confirms that shyness is a potential influencing factor for smartphone addiction.^[14]^ Researchers suggest that shy individuals are more prone to smartphone and internet addiction.^[15]^ This correlation warrants attention among medical students who require frequent social interactions.

However, the psychological mechanisms connecting shyness and smartphone addiction still require further clarification. Self-acceptance, the ability to objectively recognize and acknowledge one’s strengths and weaknesses, is a critical psychological trait influencing the mental health and interpersonal relationships of students.^[16]^ Shyness and self-acceptance are closely correlated: shy individuals often exhibit lower self-acceptance due to an excessive concern about others’ evaluations, which leads to social avoidance.^[17,18]^ This, in turn, correlates with heightened social anxiety and interpersonal difficulties.^[19,20]^ A significant negative correlation is also found between self-acceptance and smartphone addiction, with self-acceptance potentially mitigating smartphone dependence through psychological resilience enhancement.^[21–23]^ This suggests self-acceptance may mediate the relationship between shyness and smartphone addiction, with higher shyness associated with lower self-acceptance, and lower self-acceptance associated with a higher likelihood of addiction. However, this mechanism has yet to be empirically validated in medical students.

Based on the theoretical framework, this study proposes that self-acceptance, as a crucial psychological regulatory resource, mediates the relationship between shyness and smartphone addiction. Specifically, medical students with high levels of shyness tend to have lower self-acceptance due to social avoidance and negative self-evaluation, and this group is more likely to rely on smartphones for social compensation and emotional regulation, a pattern associated with a higher likelihood of smartphone addiction. Therefore, the study proposes the following hypotheses:

H1: Shyness is positively correlated with smartphone addiction;
H2: Shyness is negatively correlated with self-acceptance;
H3: Self-acceptance is negatively correlated with smartphone addiction;
H4: Self-acceptance mediates the relationship between shyness and smartphone addiction.

## 2. Method

### 2.1. Study design

This study adopted a cross-sectional survey design, collecting anonymous questionnaires through the online platform “Wenjuanxing.” This study strictly adhered to the Declaration of Helsinki. It was approved by the Ethics Committee of Yunnan University of Chinese Medicine (Approval No.: XXLW–2024–001).

### 2.2. Participants

The survey subjects were undergraduates from a medical college in China, with sampling conducted using a stratified random sampling method, stratified by grade (from freshman to senior). Each grade was allocated samples proportionally to ensure representativeness across grade levels. Data collection occurred from December 2022 to May 2023. Participants were required to read an electronic informed consent form before completing the questionnaire, which covered the research purpose, procedures, potential risks, and data confidentiality measures. Only medical students who were at least 18 years old, enrolled, and possessed personal smartphones could participate after fully understanding the information. Minors, those without smartphones, and individuals who self-reported a history of mental or neurological disorders were excluded. We collected 774 questionnaires. Except for 10 invalid questionnaires (with obvious regularity of answering, such as all items selected the same option), the remaining 8 incomplete responses were not included in the final analysis. The final effective sample size was 756, and the effective recovery rate of questionnaires was 97.67%. In addition to basic demographic information (such as gender, major, grade, ethnicity, etc), the study also collected demographic variables potentially related to key variables, such as whether the individual is an only child and family economic status, in order to incorporate the relevant demographic factors into the potential covariates for control. This study adopted the following 3 standardized Chinese scales, which have been validated as having good reliability and validity in the Chinese population, as assessment tools: the Smartphone Addiction Scale-Short Version (SAS-SV), the Shyness Scale, and the Self-Acceptance Questionnaire. Prior to administration, all scale items were reviewed for cultural sensitivity to eliminate ambiguous expressions and ensure consistent comprehension across students from different ethnic backgrounds. All data were anonymously filled out by participants, de-identified after collection, and stored in an encrypted form on a secure server.

### 2.3. Research tools

#### 2.3.1. Demographic questionnaire (self-structured)

The demographic questionnaire designed by the researchers collected baseline characteristics of participants, including grade level, specialty, gender, ethnicity, residence type, only-child status, professional identity, poverty identification, and monthly living expenses.

#### 2.3.2. SAS-SV

The (SAS-SV) was originally developed by Min Quan et al and later revised by Zhang Bin and Xiong Wanting et al It consists of 10 items rated on a 6-point Likert scale (1 = “strongly disagree,” 6 = “strongly agree”). Higher total scores indicate more severe smartphone addiction. In this study, the scale demonstrated high internal consistency, with a Cronbach’s *α* of 0.90. Previous research supports the cross-cultural applicability of this Chinese version among different ethnic groups in China, making it appropriate for the cultural sample of the present study.^[24]^

#### 2.3.3. Shyness scale

The Chinese version of the Shyness Scale, developed by Cheek and Buss, has good reliability and validity indicators. The scale consists of 13 items and uses a 5-point rating system, ranging from 1 (strongly disagree) to 5 (strongly agree), with higher total scores indicating higher levels of shyness. In this study, the Cronbach’s *α* internal consistency of the scale was 0.83, indicating high reliability. The validation study of this Chinese version among different ethnic groups in China supports its applicability to the cultural sample of this study.^[25,26]^

#### 2.3.4. Self-Acceptance scale

The self-acceptance questionnaire was developed by Cong Zhong and Gao Wenfeng. The questionnaire consists of 16 questions and comprises 2 subscales: self-evaluation and self-acceptance, with 8 items in each dimension. A 4-point scale is used, where 1 indicates “strongly opposite” and 4 indicates “strongly similar.” Higher total scores indicate higher levels of self-acceptance. In this study, the Cronbach’s *α* internal consistency of the scale was 0.75, indicating high reliability. As a localized scale, its item content and scoring method align with the cultural background of China, and validation studies involving multi-ethnic samples support its applicability to the cultural sample in this research.^[27]^

### 2.4. Statistical analysis

SPSS 27.0 was used for statistical analysis of the data. The frequency and composition ratio of general data were described. Scores for self-acceptance, shyness, and smartphone addiction were expressed as mean±standard. Pearson correlation analysis was employed to explore the correlations between self-acceptance, shyness, and smartphone addiction. Prior to conducting mediation analysis, model hypothesis testing was performed to assess the normality of core variables and the multicollinearity of diagnostic predictors. To test the mediating role of self-acceptance between shyness and smartphone addiction, and to control for the influence of potential confounding factors, the PROCESS macro developed by Hayes (Model 4) was used for analysis. In the mediation model, demographic factors that showed significant correlations were included as covariates. This was done to reduce the impact of confounding factors on the outcomes and thereby enhance the internal validity of the model. A bias-corrected bootstrap analysis with 5000 replications and a 95% confidence interval was performed to test the mediation effect, with the significance level set at *α* = 0.05.

## 3. Results

### 3.1. Common method bias

To minimize potential common method variance, several procedural remedies were implemented during data collection. First, we emphasized the anonymity and confidentiality of all responses to reduce evaluation apprehension. Second, the questionnaire instructions clearly stated that there were no right or wrong answers to encourage honest reporting. Third, the presentation order of the 3 core scales (SAS‑SV, Shyness Scale, and SAQ) was randomized for each participant using the platform’s function. Harman’s single‑factor test was also conducted to assess common method bias. The results showed that 7 factors had eigenvalues >1, and the variance explained by the largest single factor was 21.9%, which is below the critical threshold of 40%. This indicates that serious common method bias was not present in this study.

### 3.2. Demographic characteristics

As shown in Table 1, a total of 756 medical students were enrolled in this study. Socio-demographic characteristics are presented as mean±standard deviation for continuous variables and number percentage for categorical variables.

**Table 1:** Description of the distribution of the sample characteristics.

Variable	Category	N	%
Grade	Freshman	170	22.50%
	Sophomore	301	39.80%
	Junior	124	16.40%
	Senior	161	21.30%
Specialty	Nurse	427	56.50%
	Medical science	296	39.20%
	Nonmedical care	33	4.40%
Sex	Man	164	21.70%
	Woman	592	78.30%
Ethnic group	Han	544	72.00%
	Other ethnicities	212	28.00%
Household register	Countryside	618	81.70%
	Town	138	18.30%
Only child	Yes	113	14.90%
	No	643	85.10%
Do participants identify with their profession?	Yes	708	93.70%
	No	48	6.30%
Whether recognized as a financially disadvantaged student?	Yes	286	37.80%
	No	470	62.20%
Monthly living expenses (RMB/month)	<1000	158	20.90%
	1000–1500	462	61.10%
	1500–2000	112	14.80%
	More than 2000	24	3.20%

RMB = Renminbi (Chinese Yuan).

### 3.3. Correlation between variables

As shown in Table 2, the mean, standard deviation, and Pearson correlation coefficients between self-acceptance, smartphone addiction, and shyness were calculated. All 3 core variables demonstrated statistically significant correlations (*P *< .01): shyness showed a significant positive correlation with smartphone addiction (*R *= 0.312); shyness exhibited a significant negative correlation with self-acceptance (*r *=−0.621); and self-acceptance demonstrated a significant negative correlation with smartphone addiction (*r *= −0.301).

**Table 2:** Correlation of smartphone addiction, shyness, and self-acceptance ((x ± s), *r*).

Variable	Score	Acceptance	Shyness	Smartphone addiction
Self-acceptance	38.69 ± 5.73	1		
Shyness	40.84 ± 7.71	−.621*	1	
Smartphone addiction	35.05 ± 9.27	−.301*	.312*	1

* *P* < .01.

### 3.4. Smartphone addiction score

As shown in Table 3, the total score for smartphone addiction was 35.05 ± 9.27. There were significant differences in smartphone addiction scores among students of different grades (*F *= 5.145, *P *= .002), with sophomores scoring the highest (36.62 ± 8.64), significantly higher than other grades. Additionally, the addiction score of financially disadvantaged students (36.1 ± 9.35) was significantly higher than that of non-disadvantaged students (34.41 ± 9.17) (*t *= 2.438, *P *= .015). No statistically significant differences were observed in other demographic variables (*P *> .05). Grade and financial status were significantly associated with smartphone addiction scores; no other demographic variables showed significant associations.

**Table 3:** Score of smartphone addiction, shyness, and self-acceptance of college students (x ± s).

Demographic variables	Number of people	Self-acceptance	Shyness	Smartphone addiction
Grade				
Freshman	170	38.14 ± 5.51	40.41 ± 7.97	33.9 ± 9.41
Sophomore	301	38.82 ± 5.47	41.22 ± 7.63	36.62 ± 8.64
Junior	124	39.23 ± 6.04	40.94 ± 8.08	33.51 ± 10.21
Senior	161	38.61 ± 6.17	40.5 ± 7.35	34.53 ± 9.16
*F*		0.952	0.525	5.145
*P*		.415	.665	.002**
Specialty				
Nurse	427	38.67 ± 5.49	40.76 ± 7.45	34.9 ± 9.46
Medical science	296	38.86 ± 6.09	40.83 ± 8.13	34.94 ± 9.06
Nonmedical care	33	37.39 ± 5.53	41.97 ± 7.47	38 ± 8.43
*F*		0.973	0.378	1.75
*P*		.378	.685	.175
Sex				
Man	164	39.28 ± 5.46	39.94 ± 7.18	35.79 ± 10.13
Woman	592	38.53 ± 5.80	41.09 ± 7.85	34.85 ± 9.02
*t*		1.494	−1.687	1.143
*P*		.136	.092	.253
Ethnic group				
Han	544	38.62 ± 5.571	41.16 ± 7.70	35.22 ± 9.19
Other ethnicities	212	38.86 ± 6.14	40.00 ± 7.72	34.64 ± 9.50
*t*		−0.507	1.86	0.764
*P*		.612	.064	.445
Household register				
Countryside	618	38.5 ± 5.68	41.13 ± 7.69	35.3 ± 9.4
Town	138	39.52 ± 5.91	39.54 ± 7.74	34.39 ± 8.68
*t*		−1.89	2.196	0.929
*P*		.059	.028*	.352
Only child				
Yes	113	39.09 ± 6.66	40.08 ± 8.38	34.55 ± 9.93
No	643	38.62 ± 5.56	40.97 ± 7.60	35.14 ± 9.16
*t*		0.083	−1.132	−0.628
*P*		.422	.058	.530
Do participants identify with their profession?				
Yes	708	38.8 ± 5.667	40.86 ± 7.71	35.03 ± 9.16
No	48	37.08 ± 6.48	40.56 ± 7.90	35.44 ± 10.92
*t*		2.01	0.255	−0.296
*P*		.045*	.799	.078
Whether recognized as a financially disadvantaged student?				
Yes	286	38 ± 5.53	41.98 ± 7.78	36.1 ± 9.35
No	470	39.11 ± 5.82	40.14 ± 7.60	34.41 ± 9.17
*t*		−2.575	3.203	2.438
*P*		.01**	.001***	.015*
Monthly living expenses (RMB/ month)				
<1000	158	37.91 ± 5.67	41.46 ± 7.75	34.06 ± 10.58
1000–1500	462	38.62 ± 5.61	41.39 ± 7.65	35.40 ± 8.67
1500–2000	112	40.04 ± 6.03	39.26 ± 7.61	34.96 ± 9.42
More than 2000	24	38.88 ± 6.17	38.21 ± 6.71	35.25 ± 10.62
*F*		3.102	6.35	0.828
*P*		.026*	.001***	.479

F = Analysis of Variance statistic, RMB = Renminbi (Chinese Yuan), *t* = *t* test statistic.

* *P* < .05.

** *P* < .01.

*** *P* < .001.

### 3.5. Shyness score

As shown in Table 3, the total score for shyness was 40.84 ± 7.71. The shyness score of rural students (41.13 ± 7.69) was significantly higher than that of urban students (39.54 ± 7.74) (*t *= 2.196, *P *= .028). Additionally, the shyness score of financially disadvantaged students (41.98 ± 7.78) was significantly higher than that of non-disadvantaged students (40.14 ± 7.60) (*t *= 3.203, *P *= .001). Significant differences were also observed across monthly living expense groups (*F *= 6.35, *p* = .001), with the <1000 yuan group scoring highest (41.46 ± 7.75) and the More than 2000 yuan group lowest (38.21 ± 6.71). No significant differences were observed in shyness scores corresponding to other demographic variables (*P *> .05). Household registration, financial disadvantage status, and monthly living expenses were significantly associated with shyness levels; no other demographic variables showed significant associations.

### 3.6. Self-acceptance score

As shown in Table 3, the total self-acceptance score was 38.69 ± 5.73. Professional identity, student status as financially disadvantaged, and monthly living expenses significantly influenced self-acceptance levels. Students with professional identity scored 38.8 ± 5.67 (*t *= 2.01, *P *= .045), significantly higher than those without (37.08 ± 6.48). Non-disadvantaged students scored 39.11 ± 5.82 (*t *= −2.575, *P *= .01), significantly higher than disadvantaged students (38.0 ± 5.53). Significant differences were also observed across monthly living expense groups (*F *= 3.102, *P *= .026), with the 1500 to 2000 yuan group scoring highest (40.04 ± 6.03) and the 1000 yuan group lowest (37.91 ± 5.67). Other demographic variables showed no statistically significant differences (*P *> .05). Professional identity, financial disadvantage status, and monthly living expenses were significantly associated with self-acceptance levels; no other demographic variables showed significant associations.

### 3.7. Preliminary analyses and assumption checks

We evaluated the distribution of scores for shyness, self-acceptance, and smartphone addiction to assess normality. The results showed that the absolute values of both skewness and kurtosis coefficients for all variables were below 2, indicating acceptable univariate normality. To control for potential confounding effects, the following demographic variables were included as covariates in the mediation model: grade level, financial status, household registration, monthly living expenses, and academic major. The selection of these covariates was based on preliminary analyses indicating their significant correlation with the key study variables. We also checked for multicollinearity among the predictors. All variance inflation factor values were low, ranging from 1.008 to 1.653, which is well under the common cutoff of 5. This suggests that multicollinearity was not a serious concern in our model, and the regression estimates can be considered robust.

### 3.8. Testing of the mediation effect model

The model was constructed with shyness as the independent variable, smartphone addiction as the dependent variable, self-acceptance as the mediator variable, and grade level, household registration, major identification, monthly living expenses, and student financial status as covariates. Analysis was performed using the bias-corrected Bootstrap method. Table 4 reveals that shyness significantly predicts smartphone addiction positively (*β *= 0.378, *t* = 8.997, *P *< .001), explaining 10.9% of the variance in smartphone addiction (*R^2^* = 0.109). Simultaneously, shyness significantly and negatively predicts self-acceptance (*β *= -0.458, *t *= -21.412, *P *< .001), explaining 39.3% of the variance (*R^2^ *= 0.393). When shyness and self-acceptance jointly predict smartphone addiction, the combined model’s explanatory power increases to 12.7% (*R^2^ *= 0.127), with both variables demonstrating significant predictive effects (*β *= 0.249, *P *< .001; *β *= −0.282, *P *< .001). As shown in Table 5, the decomposition of effects demonstrated that the direct effect of shyness on smartphone addiction was 0.249 (95% Bootstrap *CI* [0.145, 0.352]); the indirect effect mediated by self-acceptance is 0.129 (95% Bootstrap *CI* [0.053, 0.200]). The two confidence intervals do not include 0, which indicates that self-acceptance plays a partial mediating role, and the direct effect and indirect effect account for 65.9% and 34.1% of the total effect (0.378) respectively.

**Table 4:** Results of regression analyses for the mediation model.

Step	Outcome variable	Predictor	*R*	*R^2^*	*F*	*β*	*t*
1	Smartphone addiction	Shyness	0.330	0.109	15.20	0.378	8.997*
2	Self-acceptance	Shyness	0.627	0.393	80.93	−0.458	−21.412*
3	Smartphone addiction	Shyness Self-acceptance	0.356	0.127	15.54	0.249 −0.282	4.710* −3.968*

N = 756. *β* = standardized regression coefficient.

* *P* < .001.

**Table 5:** Significance test and effect size of mediating effects.

	Effect value	Boot SE	Boot LLCI	Boot ULCI	Effect size
Total effect	0.378	0.042	0.295	0.460	
Direct effect	0.249	0.053	0.145	0.352	65.9%
Mediation effect	0.129	0.038	0.053	0.200	34.1%

Boot LLCI = Bootstrap Lower Limit for Confidence Interval, Boot SE = Bootstrap Standard Error, Boot ULCI = Bootstrap Upper Limit for Confidence Interval.

## 4. Discussion

This study investigates the relationships among shyness, self-acceptance, and smartphone addiction among undergraduate medical students, with a particular focus on the association between shyness and smartphone addiction and the mediating role of self-acceptance. The results demonstrate that shyness is positively associated with students’ propensity for smartphone addiction, a relationship that can be explained through the indirect effect of self-acceptance. Specifically, increased levels of shyness are associated with reduced self-acceptance, which in turn is associated with elevated levels of smartphone addiction. Given the single-institution sampling from a southwestern Chinese medical college, the findings should be interpreted with caution.

### 4.1. Relationship between smartphone addiction and shyness

The study reveals a 65% smartphone addiction rate among medical undergraduates, significantly higher than the 41.93% reported by Zhong Ying et al.^[7]^ which may be related to the intense academic pressure, demanding clinical training, and strict professional requirements of medical students that drive frequent smartphone use and reliance.^[22]^ These findings underscore the seriousness of smartphone addiction in this sample and provide practical evidence for targeted interventions. Notably, the high addiction rate and shyness-addiction correlation observed are specific to this single sample and may not reflect the situation of medical students in other regions or institutions. The study also found that shyness was positively associated with smartphone addiction, which aligns with earlier research.^[14]^ Notably, participants scored relatively high on the shyness scale. Shyness is characterized by social anxiety, excessive worry about negative feedback, and social avoidance tendencies,^[28]^ and the strong social skills required for medical education (e.g., case discussions, simulated clinical practice) and future clinical careers (e.g., doctor-patient communication) make shyness associated with feelings of frustration and unease in real-world interactions. While smartphones offer a low-pressure virtual space for shyness, prolonged reliance on this channel is associated with a poorer ability to handle real-world social challenges, a pattern further correlated with higher smartphone dependence. For medical students, a high-stress group, this cycle may be intensified by the dual pressures of academic and professional demands.

This study reveals a significant correlation between shyness and smartphone addiction, suggesting that shyness is an internal psychological trait associated with the development of core communication skills in medical students. These findings provide a basis for identifying at-risk groups, conducting early psychological screening, and delivering targeted support. Addressing the shyness and smartphone usage behaviors of medical students is a crucial step in promoting their holistic development. Medical schools should enhance mental health education through group counseling and social skills training to help students improve their self-acceptance and social confidence while guiding them toward responsible smartphone usage. For students with severe issues, timely professional psychological support should be provided.

### 4.2. Mediating role of self-acceptance

This study reveals that self-acceptance mediates the relationship between shyness and smartphone addiction. Higher shyness is associated with lower self-acceptance, which in turn is associated with higher smartphone addiction tendencies. This finding enriches the research framework on factors influencing smartphone addiction and provides a new theoretical perspective for understanding the intrinsic connection between shyness and addiction. The results align with the core concept of self-cognition theory: self-acceptance, as a fundamental component of positive self-cognition, reflects an individual’s acceptance and recognition of their own traits. ^[29]^ Shy individuals often exhibit negative self-cognitive biases, focusing excessively on their social shortcomings, a pattern associated with self-denial and inferiority complex.^[28]^ When self-acceptance is low, individuals struggle to alleviate social anxiety through internal psychological regulation and tend to seek emotional compensation from external sources. The virtual environment of smartphones offers a “low-risk self-presentation” space where individuals can filter information and edit expressions to avoid real-world social exposure risks, obtaining instant social feedback and value recognition. This compensatory use of smartphones is associated with a higher likelihood of addictive behavior.^[30]^ This study likewise expands the “shyness to self-acceptance to addiction behavior” mediating chain model, providing a theoretical framework for future cross-group and cross-cultural research.

Validating the mediating effect of self-acceptance identifies a key target for interventions aimed at reducing smartphone addiction among medical students. Compared with shyness, a relatively stable personality trait that can be difficult to address directly, self-acceptance is a psychological quality that can be enhanced through psychological interventions. Accordingly, medical schools can offer mental health courses and group counseling sessions to help students objectively recognize their own traits, cultivate self-tolerance and self-acceptance, and in turn reduce the impact of negative self-cognition on their behavioral decisions. For example, with mindfulness training and cognitive restructuring techniques, shy students can lower their perfectionist expectations for social interactions, build greater psychological resilience when dealing with social challenges, and thus reduce excessive reliance on virtual social connections.

### 4.3. Students identified as financially disadvantaged exhibited significantly higher levels of shyness, smartphone addiction, and lower self-acceptance among medical students

This study further reveals that poverty status significantly influences the shyness, self-acceptance, and smartphone addiction of medical students. These findings align with research demonstrating the close relationship between economic stress and mental health behaviors.^[31,32]^ It is further noteworthy that this correlation originates from multiple intertwined factors. Practically, financially disadvantaged students face hidden social activity hurdles due to limited resources, such as inability to cover group event costs and social self-consciousness from material differences. These resource barriers are correlated with social avoidance, and long-term social withdrawal is associated with higher shyness tendencies.^[33]^ Psychologically, long-term economic deprivation is associated with lower self-worth through social comparison, a pattern further linked to weaker self-acceptance.^[34]^ The combination of higher shyness and lower self-acceptance is associated with a “social avoidance-self-denial” cycle, which is correlated with reduced willingness and ability to engage in real-world social interactions. In this context, low-cost and accessible smartphones serve as important tools for financially disadvantaged students to alleviate psychological stress and meet social needs: online entertainment relieves real-life negative emotions, and digital social platforms avoid in-person interaction awkwardness while providing instant emotional support and a sense of belonging. But without proper regulation, this “online compensatory use” is associated with excessive reliance on smartphones, which in turn is correlated with a higher likelihood of smartphone addiction.

This finding suggests that financial disadvantage status is a demographic factor associated with shyness, self-acceptance, and smartphone addiction among medical students, though these effects are not absolute but rather shaped by multiple factors. Therefore, when addressing challenges faced by economically disadvantaged students, it is crucial to holistically consider various factors and develop targeted support and intervention measures. These efforts will help them cultivate positive self-perception, enhance stress management skills, and reduce psychological issues and behavioral tendencies stemming from financial circumstances. The observed associations between financial disadvantage and the 3 core variables are limited to this sample, and regional economic and institutional differences may lead to different results.

## 5. Conclusion

This cross-sectional study provides evidence that self-acceptance partially mediates the association between shyness and smartphone addiction among medical students. This finding offers a new perspective for understanding the psychological mechanisms behind such addiction and provides theoretical support for developing targeted mental health interventions. Several limitations warrant attention: the cross-sectional design prevents causal inferences with the proposed mediation pathways needing validation through longitudinal or experimental studies; the convenience sample from a single medical school restricts the generalizability of results; and reliance on self-reported data may introduce common method bias. Future research should use longitudinal or experimental designs with larger multi-center samples to verify causal relationships, and incorporating objective measures will enable more rigorous examination of these associations to refine relevant theories and inform intervention practices.

## Author contributions

**Data curation:** Zhimin Yin, Rong He, Qin Liu.

**Formal analysis:** Zhimin Yin, Rong He, Qin Liu.

**Methodology:** Zhimin Yin, Renjie Chang.

**Software:** Zhimin Yin, Qin Liu.

**Conceptualization:** Rong He, Qin Liu.

**Funding acquisition:** Rong He, Renjie Chang, Qin Liu.

**Project administration:** Rong He, Renjie Chang.

**Supervision:** Renjie Chang, Qin Liu.

**Validation:** Renjie Chang, Qin Liu.

**Writing – original draft:** Zhimin Yin, Rong He.

**Writing – review & editing:** Zhimin Yin, Qin Liu.
